# Workplace factors that promote and hinder work ability and return to work among individuals with long-term effects of COVID-19: A qualitative study

**DOI:** 10.3233/WOR-220541

**Published:** 2023-08-11

**Authors:** Kristina Gyllensten, Alexander Holm, Helena Sandén

**Affiliations:** a Department of Occupational and Environmental Medicine, University of Gothenburg, Gothenburg, Sweden; b Sahlgrenska University Hospital, Gothenburg, Sweden

**Keywords:** Long COVID, work conditions, need for recovery, qualitative research

## Abstract

**BACKGROUND:**

Long COVID is defined by the persistence of physical and/or psychological and cognitive symptoms debuting after SARS-CoV-2 infection. Individuals affected describe impairing and debilitating symptoms sometimes making it difficult to take part in work and social life. Long COVID is likely to have an impact on the work force.

**OBJECTIVE:**

The aim of the study was to explore workplace factors that promote and hinder work ability and return to work among individuals with long-term effects of COVID-19.

**METHODS:**

A qualitative design was used. Data were collected by semi-structured focus group interviews and analysed using inductive thematic analysis. To increase trustworthiness, several researchers were involved in the data collection and analysis. Five focus group interviews were conducted with individuals suffering from long-term effects from COVID-19 affecting their work ability. In total, 19 individuals participated in the study, and all were working at least 50 per cent at the time of recruitment.

**RESULTS:**

Five main themes emerged from the analysis: Communication and support, Possibilities to adjust work, Acceptance of new limitations, Increased need for recovery from work and Lack of knowledge and understanding of the effects of Covid.

**CONCLUSION:**

The results suggested that it is useful to facilitate communication, support and work adjustments for individuals suffering from Long COVID. It is also important to accept limitations and fluctuations in work ability and encourage recovery during and after work.

## 1 Introduction

Long COVID is defined by the persistence of physical and/or psychological and cognitive symptoms debuting after SARS-CoV-2 infection, often three months after the acute infection, and lasting more than three months [1, 2]. Different definitions are used in the literature, and the World Health Organization [3] uses the term post-COVID-19 condition [3]. It has been proposed that Long COVID may not be a single syndrome, but several syndromes, such as post-viral fatigue syndrome and post-intensive care syndrome [2]. Individuals affected describe complex, impairing and debilitating symptoms sometimes making it difficult to take part in work and social life [4], and common symptoms include fatigue, breathing difficulties, headache, chest pain, smell and taste disturbances, brain fog and memory loss and sleep disorders [1, 5].

Long COVID is likely to have an impact on the work force, and an umbrella review of the long-term consequences [1], including 23 reviews and 102 studies, found that in previously hospitalized cases 9–40% reported absence from work due to Long COVID at two to three months after discharge. In mild to moderate non-hospitalized cases, it was found that 12%–23% remained absent from work three to six months after acute disease, and apart from full absence, studies reported that many suffering from Long COVID had to adjust or reduce their workload. The authors concluded that Long COVID will likely have a substantial public health impact, although the current evidence is incomplete and heterogeneous [1]. A Swedish national registry-based study of 11 955 individuals on sick leave for Covid found that 13.3% were on sick leave for Long COVID and that this group appears to be heterogeneous and should not be neglected [6]. Another study investigated the impact of post-Covid syndrome on functioning in 1027 patients in Germany after mild and moderate SARS-CoV-2 infections. It was found that long-term symptoms are common and lead to limitations of activities, but that in most cases the limitations are not severe and do not lead to serious issues with work ability or quality of life [7]. The European Agency for Safety and Health at Work [8] have published a discussion paper regarding the impact of Long COVID on workers and workplaces, and write that Long COVID presents a considerable challenge for employers because important workers could have difficulty returning to their ordinary jobs within the normal timescales. They suggest that important issues to consider are what adjustments can be made to the job or to the working hours, what support is needed and what the functional limitations of workers with Long COVID are, and conclude that further research is needed to improve the knowledge on workplace issues related to Long COVID.

The concept of work ability is multidimensional and involves the physical, psychological and social capability of an individual to perform and interact with their work, and the individual’s work demands, health and resources [9, 10]. Previous research has found that a number of factors influence work ability among individuals with long-term diseases, including job demands, age, gender and somatic complaints [11].

It has been argued that in order to overcome remaining uncertainties regarding Long COVID it is important that future research should include methodologically sound epidemiological studies and that these should be complemented by qualitative studies that capture the experiences of individuals with Long COVID [1]. It was also suggested that to fully understand such multifaceted and complex health conditions, approaches that capture and amplify the voices of those affected are needed. There have been a few qualitative studies investigating the experiences of Long COVID [12–14], but to our knowledge none that explore factors influencing work ability and return to work. Considering, the fact that Long COVID appears to have a negative effect on work ability, exploring individual accounts of Long COVID and work-related factors can provide valuable insights. Therefore, the aim of this study was to identify workplace factors that promote and hinder work ability and return to work among individuals with long-term effects of COVID-19.

## 2 Method

### 2.1 Background

The study was part of a larger research Interreg project titled ‘Behandling af COVID-19 Senfölger’, financed by the European Union. This was a collaboration between different research institutions in Sweden and Denmark. At our institution there has also been a quantitative study investigating the effects of COVID-19 on small airways.

### 2.2 Participants

A purposeful sampling strategy was used to ensure that the sample consisted of participants with relevant experience relating to having long-term effects of COVID-19 affecting their work. The participants were recruited via two different routes. One route was via the quantitative study, at our institution, investigating small airways. All participants from this larger study who reported that they were working at least 50% and had long-term effects after their COVID-19 infection influencing work were invited to participate in the study. COVID-19 infection was verified by a positive SARS-CoV-2 ribonucleic acid (RNA) real-time polymerase chain reaction (RT-PCR) test via a nasal swab. The other route of recruitment was via post-Covid clinic at a large University Hospital in the southwest part of Sweden. Patients who had attended the clinic during the last couple of months and who were working were invited via a letter to participate. In total, 19 individuals participated in the study; all were working at least 50 per cent at the time of the recruitment and had long-term effects from Covid that were affecting their work ability. They received no reimbursement for their participation. Background information on the participants is presented in Table 1.

**Table 1:** Information regarding participants and focus groups

Occupations	Teacher (2), web designer, welder (2), administrator, psychotherapist, engineer, manager (2), cleaner, nurse (4), caretaker, assistant nurse (2), finance assistant
Gender	6 males, 13 females
Age	29–63 years, mean age 54
Live interviews	3
Digital interviews	2
Recruited via study on airways	13
Recruited via post-Covid clinic	6
Number of participants in each focus group	4, 3, 4, 5, 3
Months since Covid	median 18, mean 15, range 4–22
WAI score	median 6, mean 5,9, range 3–10
Physical symptoms of Long COVID	Impaired memory and concentration, brain fog, fatigue, respiratory symptoms, pain, sound sensitivity

### 2.3 Procedure and data collection

The data were collected using semi-structured focus group interviews. This approach allows for flexibility during the interviews and a sharing, collaborative discussion of different experiences and opinions between participants [15]. Interviews were performed from November 2021 through April 2022. The interview guide, which had been developed in relation to the research aim and existing literature, contained open questions about experiences of long-term effects from COVID-19 at the workplace. Follow-up questions were used to encourage the interviewees to elaborate, in order to reach a deeper understanding [16]. Examples of questions included: ‘What at work has made it more difficult/easy to return to work?’ ‘What factors have had a positive/negative effect on your work ability?’

Five focus-group interviews were conducted. Three of the interviews were conducted at the University Hospital where the researchers were located and two of the interviews were conducted digitally. All interviews were planned to be conducted live, but due to the fact that new Covid restrictions were implemented at the hospital in December 2021 it was not possible to invite participants to do the interviews live. The digital interviews did not differ in content or length compared to the live interviews. Participants in the focus groups did not know each other. The interviewers paid close attention to the group dynamic and existing informal or formal power relationships [15]. Four of the interviews were conducted together by KG and HS and one was conducted by KG and AH. KG had prior experience of running focus groups. The interviews lasted approximately one hour each. Interviews were tape-recorded and professionally transcribed verbatim. The quotes used to support the analysis in this paper were translated from Swedish to English by a professional translator after the analysis. Questionnaires for assessing work ability score and sickness absence were also conducted. The question about work ability, was communicated as work ability score (WAS). WAS is a self-assessment of current overall work ability level in comparison to lifetime best and it ranges from 0 to 10 [9, 17].

### 2.4 Data analysis

A qualitative method was used, as this was deemed suitable to explore the experiences of factors that promote and hinder work ability and return to work among individuals with long-term effects from COVID-19. More specifically, inductive thematic analysis was used to search for themes in the data, in accordance with the method described by Braun and Clarke [18]. Thematic analysis is theoretically flexible, which means that it can be applied using different epistemological and ontological approaches, in contrast to methods bound to specific theoretical frameworks such as grounded theory. In this study a realist approach was adopted, as the aim was to explore the participants’ experiences of their reality via interviews, rather than attempting to interpret the social constructs or values that may have an effect on these experiences [18, 19]. An inductive, data-driven approach was chosen, as that would enable the identification of unexpected themes, and would provide a rich, multifaceted and detailed account of the underlying data, which was not preconceived by us as researchers. However, a reflexive attitude was adopted, and critical discussions were used to attend to the context and consider our pre-existing knowledge. In an inductive, data-driven approach, the resulting themes are strongly linked to the data. Thus, coding is performed without a predefined framework and without engaging with existing literature during the analysis [16]. It was assumed that the investigation of factors influencing work ability and return to work could result in a number of different themes, rather than being explained by one single phenomenon. Hence, the aim was to present a description of the entire dataset relating to the research topic. This is appropriate when there is little previous research and knowledge on the topic, as described by Braun and Clarke [18]. This was also a reason why the inductive thematic analysis was considered more suitable than, for example, grounded theory.

The initial coding and analysis was done by the first author (KG) in accordance with the six phases described by Braun and Clark [18]. The first and second steps were to become familiar with the data by reading each transcript several times, while registering initial codes that captured interesting features of the data. The entire dataset was systematically coded. In a third step, emerging conceptual themes were identified. Themes were identified throughout the process based on their ‘keyness’, described as capturing something important in relation to the research question [18]. In the next step, the list of main themes was reviewed and refined until a final list of clearly defined main themes and subthemes was established, capturing coherent data to create mutually exclusive themes. This step meant reading the coded extracts within each theme to determine the coherence of themes and find patterns while staying close to the data. To strengthen trustworthiness and inter-rater reliability, one of the co-authors (HS) also read all the interviews and checked the coding done by the first author. KG and HS discussed and revised the themes and reviewed the extracts until a final list of main themes and subthemes was agreed on. Finally, the themes were named and defined, and specific quotes from the interviews were chosen to capture and illustrate the essence of each theme. See Table 2 for an example of the analytical process.

**Table 2:** An example of the process of abstraction

Unit of analysis	Code	Subtheme	Main theme
*For example I may say* ‘*I need to go out for a walk*’*, and then everybody knows that I will be gone for half an hour. And I know that they will check on me:* ‘*Is she coming back? How does she look*’*? So, it feels good. They have, they are silently looking out for me* ... *it is really good.*	Experience a silent social support from the colleagues. This support is much appreciated.	Communication and support from colleagues	Communication and support

### 2.5 Ethics

This study was approved by the Ethical Review Board, Sweden (2020–05681). Informed consent was obtained with an information letter and the signing of a consent form. Information included, among other items, that participation was voluntary, that participants could withdraw at any time without having to give a reason and that the results would be presented in an anonymized form.

## 3 Results

The analysis of the data resulted in identification of five main themes and a number of subthemes (see Table 3). Three of the main themes were factors promoting work ability and returning to work, and these were Communication and support, Possibilities to adjust work, and Acceptance of new limitations. The other two main themes were hindering factors, and these were Increased need for recovery from work, and Lack of knowledge and understanding of the effects of Covid.

**Table 3:** Main themes and subthemes

Main themes	Subthemes
Promoting factors
1. Communication and support	1.1 Communication and support from management
	1.2 Communication and support from colleagues
2. Possibilities to adjust work	2.1 Working from home
	2.2 Flexible working hours
	2.3 Adjusted work tasks
	2.4 Long-term need for adjustment
3. Acceptance of new limitations	3.1 Difficult to decrease work pace
	3.2 Acceptance of lower work ability
	3.3 Individual strategies
Hindering factors
4. Increased need for recovery from work	4.1 Lack of energy at work
	4.2 Taking breaks at work
	4.3 Overly tired after work
5. Lack of knowledge and understanding of the effects of Covid	5.1 In the workplace
	5.2 In society

### 3.1 Theme 1 - Communication and support

The participants expressed that communication and support were very important both for returning to work and for having a good work situation. These factors were important in relation to communication both with the managers and with colleagues.

#### 3.1.1. Communication and support from management

Good communication and sufficient support from the managers were viewed as almost necessary for being able to have a reasonable work situation while suffering from long-term effects of COVID-19.

*But I will continue to be clear with my manager and tell her how I feel, and she is really good at asking. If I get tired it is ok for me to lie down, regardless of where I am, really, and that has been a strange experience, that I really have to switch off and lie down.*

*She has done a lot, my manager*  ...   *she has told my colleagues* ...  *Yes, she has said that I don*’*t have any specific time I have to tend to. They have to be forgiving regarding that.*

Approximately half of all participants reported that they had good communication with their managers regarding their health and their needs following Covid, but this was not the case for all. Some participants clearly expressed that they wanted more communication at the workplace.

*I think that they don*’*t talk about it enough. They don*’*t ask me how I am feeling, how I have been, there are no follow-ups.... Nothing is done really. They don*’*t ask how the employee is feeling, and still, we have, there are lots of us that have gone to occupational health services, lots.*

*My previous employer asked pretty regularly, how are you feeling, how is it going, are you seeing a doctor? And so on, it was very good. But the new one, she has hardly asked me anything about it, even though she knows, and then we have these employee talks every other week.*

#### 3.1.2. Communication and support from colleagues

The relationship with the colleagues was also very important for having a good work situation. In some instances, the colleagues had volunteered to take over the more demanding work tasks in order to decrease the work demands for the participants.

*But the days that I feel that, no today is not a good day, I have wonderful work colleagues, so I am able to swap work tasks.*

Being able to discuss symptoms with colleagues was viewed as very positive by some. Others felt that their colleagues silently were watching out for them to make sure that they were feeling all right, and this provided a sense of being caredfor.

*For example, I may say* ‘*I need to go out for a walk*’ *and then everybody knows that I will be gone for half an hour. And I know that they will check on me,* ‘*Is she coming back, how does she look*’*? So, it feels good. They have, they are silently looking out for me*  ...   *it is really good.*

One participant described having been very open about her symptoms and getting a lot of support from colleagues.

*I have received a lot of support because I have been pretty open.*

### 3.2 Theme 2 - Possibilities to adjust work

Having been able to adjust various aspects of the work situation had been crucial for many participants. Working from home, flexible work hours and adjusting specific work tasks were examples of this. However, some participants expressed that it was a problem that certain adjustments were only done short-term.

#### 3.2.1. Working from home

Being able to work from home had been a basis for being able to work for many participants. This had made it possible to get more recovery during the workday.

*And it is really important with support from the employer.* ...   *I receive great support, and together we have decided that I am allowed to work from home two days a week in order to save some energy getting back and forth to work. So, I feel an amazing support and I am very grateful for that*  ...   *because otherwise it would probably have been pretty impossible to work those 80 per cent that I work.*

Working from home also had the advantage of having a better sound environment with fewer disturbing sounds.

*I have worked from home a lot, because then I can escape the murmur around me, and we are in an open plan office. So that makes it easier*  ...   *but at the same time I miss my colleagues, and the social part.*

#### 3.2.2. Flexible working hours

Flexible working hours were also really helpful according to the participants who had this opportunity, as this gave them the chance to plan the day to get sufficient recovery.

*So, I can divide my working hours during the day, and that is an advantage for me. So perhaps this is the reason to why I can do my job. Because I have an incredible freedom in how I plan my time two days a week.*

#### 3.2.3. Adjusted work tasks

Another possibility that was very helpful was adjusting work tasks, for example, being able to alternate between different tasks or doing less demanding tasks.

*Luckily, I can switch from assembling and welding.*

*The employer has been very accommodating, so my colleagues do some of my work and I have done a little more administrative tasks.*

Some participants said that their managers had suggested that some tasks should be removed in order to decrease the workload, and this was viewed as very positive.

*I am a first aid instructor among other things. We have different tasks apart from our ordinary work, and it was my manager who said that if I wanted, we could remove, so that I would have sufficient energy for work. And I experienced that as very positive, actually.*

However, not everyone had the possibility of adjusting or removing work tasks or having flexible working hours, and this was highlighted as an example of where there was a lack of understanding from the employer.

*There was a training day in the middle of it all, yes,* ‘*You only have yourself to blame if you don*’*t learn during the training day,*’ *but I don*’*t have the energy.* ...   *So, the employers*’ *adaption is not an adaptation to me, it is more like,* ‘*If you can*’*t work, call in sick.*’ *So, you get the option of sick leave thrown in your face all the time.*

#### 3.2.4. Long-term need for adjustment

Since the effects of Covid were long-term, it was important that the support and adjustment was long-term. And several participants experienced that this support had been available from the start but had been withdrawn after a period of time. This was described as a gradual process.

*They promised me that I should be able to take it easy, and that they should remove work tasks from me*  ...   *and that I will do the things I have the energy to do.* ...   *But a week went by and then the work was building up more and more and more. And now I am back to the old ways again, although I don*’*t have the energy.*

*So really, I don*’*t think they have done much wrong. It is more that I think that they forget, because it does not show, so they don*’*t think about the fact that I am still ill*  ...   *and they give me as much to do as they give the others.*

### 3.3 Theme 3 - Acceptance of new limitations

The participants described new physical and psychological limitations following the COVID-19 infection that affected their work ability. However, for some it was difficult to decrease the work pace, despite feeling that they needed to slow down. A part of accepting the new limitations was to create practical strategies to handle various demanding situations.

#### 3.3.1. Difficult to decrease work pace

Some participants expressed that they worked more than they ideally should, considering their fatigue and lack of energy. They felt that despite the fact that a high work pace would lead to negative health effects, it was still difficult to slow down the pace.

*And I know that sometimes I do more than I should, because my body protests. It is a little stupid, but what should I do?*

*Maybe I should not have increased my working hours up to 75 per cent, because I can*’*t really handle more than 50 per cent.*

There was a need to find a good balance between activity and rest.

*So, I have to be in some kind of in between mode to feel ok. It is not possible to go all in, or not go at all.*

#### 3.3.2. Acceptance of lower work ability

Many in the group expressed that it was very difficult to accept the fact that they now had a lower work ability compared to before COVID-19. Giving up certain work tasks because of the lower work ability and accepting impaired work performance because of the effects of COVID-19 was a challenge.

*I have been pretty active with supervision, etc., but I had to drop these parts. It was very, very difficult*  ...   *not being able to perform in the same way as I did before.*

Part of the process of acceptance was to listen to the body’s signals and to rest when this was needed. For some it was very difficult to accept the decreased work ability, and there was a strong wish to be the same as before the COVID-19 infection. Several participants described a sadness related to being different and having a lower work ability compared to previously.

*And I was never off sick, so there is something wrong in my head. I have some difficulty in adapting and accepting. Now, it will have been two years soon. And this is the way it is, you have to adapt your life according to this instead.*

*And I want, I want so badly to be a fully functioning human being the way I used to be, but yes, I am someone else, a pale copy, a shadow of myself, in every way.*

#### 3.3.3. Individual strategies

There were different examples of practical strategies that the participants were using to compensate for the new limitations. Examples included writing things down when the memory worsened.

*Now I have to write down everything exactly, and I prefer mail to phone calls, so that I have everything in writing.*

### 3.4 Theme 4 - Increased need for recovery

The participants reported feeling more tired and having an increased need for recovery following the COVID-19 infection, which highlighted the increased need for recovery both during and after the working day. Taking breaks during the workday was one way of handling the increased tiredness.

#### 3.4.1. Lack of energy at work

The fatigue built up during the workday, and many described feeling very tired both during work and after work. Some experienced the fatigue as crippling and found it very difficult to continue to work as they normally did when experiencing this fatigue.

*It is possible, but I have to mobilize myself quite a lot to cope. But I manage to work five to six hours, but then it is like you say, it feels like I will collapse. And if I push myself, no then I will faint.*

#### 3.4.2. Taking breaks at work

Taking breaks during work was an important strategy to get sufficient recovery during the workday.

*I take pauses. It is the way I have tried to handle it. Then some days are better, and other days are worse, so I can feel alert, and then an hour later I don*’*t want to be there at all.*

For some it was difficult to find sufficient recovery at work and this led to the participants becoming very tired after work.

*There is no time to sit down and relax, I don*’*t have that at all. So, there is no room for rest, and I think that is the reason to why I go home and crash, just as you describe it.*

#### 3.4.3. Overly tired after work

Many reported an increased need for recovery after work and described feeling so tired that they ‘crashed’ when they got home from a workday. Thus, a big part of their daily energy was consumed during work, which meant that there was very little energy left for the other parts of life.

*I just wanted to concur that the little energy I have got, I spend it at work. And when I come home there is no energy left for the other parts of life. And I think that is distinctive for many of us, that we have this feeling of responsibility and that we so badly want to come back.*

*I have managed to work, but then I have not managed much more.* ...   *I come home and kind of die. But it has worked, and in a way it has been good to focus on something other than myself.*

### 3.5 Theme 5 - Lack of knowledge and understanding of the effects of COVID-19

According to the participants, they had been negatively affected by the lack of knowledge of the effects of COVID-19, both at the workplace and in society as a whole.

#### 3.5.1. At the workplace

Several participants experienced that their managers lacked understanding regarding the needs of the workers who suffered from long-term effects of Covid. According to some participants, they did not expect that the managers should be able to do anything about their health, but they wished that they would show empathy.

*Many times, words would be enough. They cannot do anything about my symptoms, but they can show compassion. I mean about knowing that I am ill because of Covid.*

One participant described how she felt questioned by the Human Resources department.

*And I end up in these meetings with HR because I had to be off sick, where my work ability is questioned. And that feels hard because I was infected at work. I couldn*’*t help it.*

It was suggested that it was important to improve the understanding of the effects of COVID-19 among managers.

*I think it is very important that the managers are aware of what we have been through, because there could be someone else that gets the same thing.*

#### 3.5.2. In society

According to the participants, there was also a lack of knowledge of the long-term effects of COVID-19 in greater society. And this could make some feel vulnerable.

*Because right now, it is a cultural disease and the related debate. And it is strange for us, because I feel very vulnerable. And there is nothing I want more than to be an active, fully functioning human being in the society we live in.*

## 4 Discussion

The current study aimed to explore factors that promote and hinder work ability and return to work among individuals with long-term effects of COVID-19. Five main themes emerged from the inductive thematic analysis, and three of these highlighted factors promoting work ability and return to work: Communication and support, Possibilities to adjust work and Acceptance of new limitations. Two of the themes highlighted hindering factors: Increased need for recovery from work and Lack of knowledge and understanding of the effects of Covid. Below, each theme will be discussed in turn.

The first main theme was *Communication and support*, which related to communication and support from both management and colleagues. When the communication worked well, it was one important reason that it was possible for the participants to work, despite having disabling symptoms. Thus, good communication with management and colleagues facilitated work ability. Conversely, when the communication and support were missing, the participants described this as a hindering factor at work. Supportive behaviour included management informing other employees about the situation and enabling flexibility regarding work times and tasks, and colleagues offering to do the more demanding work tasks when the participants were struggling. This result is similar to the guidance on Long COVID for managers proposed by EU-OSHA [20] that states that regular discussions between the worker and managers is important in the rehabilitation of workers with Long COVID, and that trust between managers and employers is important for effective adaptation of work. Further, EU-OSHA states that the manager does not have to be an expert in Long COVID, but that they should listen to concerns and provide support to the worker with Long COVID. Social support meets basic human needs of group membership and companionship [21], and support at work has been described as the product of interpersonal work relationships that have the potential to promote well-being and coping [22]. Support is a central factor in the most influential theoretical model of psychosocial stress at work, the demand–control–support model (JDC-S model) [23], where social support has a buffering effect [24]. Previous research has found that social support from supervisors and colleagues buffers the effects of job stress on physical and psychological well-being, and can play a role in employee effectiveness [22, 25]. Good communication, which can be seen as a form of supportive behaviour, has also been put forward as an important factor that can influence well-being at work [25].

The second main theme was *Possibilities to adjust work*. The participants described various forms of adjustments that were helpful, such as being able to work from home, and having flexible working hours and adjusted work tasks. However, it was important that the adjustments were maintained long-term and not removed after a few weeks. Once again, the importance of good communication with the managers was highlighted. Previous research has also found that Long COVID affected work. In the umbrella review by Nittas et al. [1], it was reported that many suffering from Long COVID had to adjust or reduce their workload, and it was concluded that Long COVID will likely have a substantial public health impact. Two cohort studies with previously hospitalized participants found that 15% and 40%, respectively, adjusted their employment in accordance with their current circumstances [26, 27]. The EU-OSHA [20] proposes that employers should consider what adjustment can be made to the job or the working hours of individuals with Long COVID.

Regarding developing structured methods for rehabilitation for workers suffering from Long COVID, it could be useful to learn from existing rehabilitation programmes. Indeed, Godeau [28] suggested that there is a need to implement strategies promoting return to work for these workers, and that these programmes could be similar to those developed for other chronic conditions. Similarly, previous research not related to Covid has suggested that flexible working arrangements are important to reduce stress and increase mental well-being at work [23].

*Acceptance of new limitations* was the third main theme. All participants suffered from symptoms that affected their work ability in various ways, and they reported that it was very important to accept this change. Some found acceptance almost impossible, as they wanted to perform in the same way as before they became ill, and others reported that they had come quite far in the process of acceptance. Previous research has reported different symptoms of Long COVID that can have an impact on work performance, including fatigue, breathing difficulties, headache, chest pain, smell and taste disturbances, brain fog and memory loss and sleep disorders [1, 5]. In addition, Long COVID can also cause absence from work, and Nittas’s [1] umbrella review found that 9%–40% reported absence from work due to Long COVID at two to three months after discharge in previously hospitalized cases. As well, 12%–23% remained absent from work three to six months after acute disease, in mild to moderate non-hospitalized cases. A previous qualitative study of 24 individuals suffering from Long COVID found that the persistent problems following Covid had changed them as individuals and were a threat to their identity [13]. It has been suggested that scales such as WAI can be useful in the assessment and follow-up of patients with Long COVID as this can provide important information about work capacity and workload [28]. In the current study all participants completed WAS score [9, 17] and the mean participants’ scores were lower than the scores of the general working population [29]. This result fitted well with the information gained from the interviews.

The fourth main theme was *Increased need for recovery from work*, and the participants described having a continuing high need for recovery during and after work. The work tasks were ‘costing’ more energy-wise; nevertheless, the participants did their best to perform their work in the same manner as before they became ill. Indeed, energy is a limited resource that varies each day within individuals [30]. Work requires energy and effort, and after expending energy over a period of time, it is necessary to replenish resources and recover [31]. The concept need for recovery (NFR) describes the requirement to physically and mentally recuperate following a period of work [32, 33]. If an individual does not get sufficient recovery, and the NFR cumulatively increases, there is a risk for a negative impact on physical health, psychosocial well-being and occupational performance [33]. An ongoing high NFR is related to a variety of long-term negative health effects, including depression, cardiovascular disease and sickness absence and psychosomatic symptoms [34–37]. The participants in the current study described using most of their energy during work, and having little or no energy after work. The participants’ work demands may not necessarily have been very high in a general sense, but they appear to be too high, considering their high need for recovery. It could therefore be suggested that it is important that individuals suffering from Long COVID get sufficient recovery during and after work, and that the work demands are assessed and adapted in accordance with their current (and changing) health status, NFR and workability.

The final main theme was *Lack of knowledge and understanding of the effects of Covid*. According to the participants, there was a lack of understanding both in the workplace and in the general society. The participants with Long COVID in the previously mentioned qualitative study [13] stated that in relation to recovery and rehabilitation it would help to be listened to, believed and understood. Another qualitative study of doctors with Long COVID found that the participants needed to use their knowledge and connections and self-advocate, as there was a lack of knowledge regarding how to manage their symptoms [14]. In a discussion paper regarding the impact of Long COVID on workers and workplaces, the EU-OSHA [8] proposes that employing organizations’ normal sickness absence policies may have to be revised and timescales extended for workers with Long COVID, as recovery may be very slow. They suggest that most workers recovering from Long COVID will require a phased return to work with gradually extended work hours for months.

### 4.1 Implications

An important question is whether there is anything specific that needs to be considered in relation to return to work and work ability for individuals suffering from Long COVID. There is, of course, much previous knowledge regarding rehabilitation for other disabling conditions, and it is possible that much of this can be adapted to this group of patients, as suggested by Godeau [28], who also suggests that there is a need for enhanced support by occupational health and rehabilitation specialists for individuals suffering from Long COVID. Based on the findings from the current study, the following implications are suggested:

• Facilitate and encourage continuous communication with individuals suffering from Long COVID and discuss work-related consequences of Long COVID. The communication does not necessarily have to be complex or based on any specific structure; instead, basic questions showing interest and care for the individual can be perceived as very supportive.
• Facilitate adjustment of work, for example, flexible work hours, working from home and adapted work tasks. If this is not possible, have a dialogue with the employee regarding this fact and discuss whether there are small things that can be adjusted.
• Accept the new limitations of individuals suffering from Long COVID and support them in finding acceptance. Be open to a fluctuating work ability. Thus, assess and if necessary, adapt the work demands accordingly.
• Encourage and facilitate recovery opportunities at work, including regular breaks and shorter pauses.
• Help to battle the potential stigma of Long COVID by talking about it. There is a general lack of knowledge regarding Long COVID, and it is therefore reasonable that most people at the workplace know very little or nothing at all about the topic. Nevertheless, it is still possible to provide support and to be open to the wealth of new knowledge that is likely to come within the near future.

Future research, both qualitative and quantitative, should further investigate what issues influence work ability and return to work among individuals suffering from Long COVID.

### 4.2 Strengths and limitations

It is possible that the participants found it difficult to fully express their opinions in the focus groups. The interviewers informed the groups that all opinions, experiences and ideas were welcome, and there was no need for consensus in the group. Another limitation is that two of the focus groups were conducted digitally, and it is possible that the digital format inhibited the participants’ discussions. However, the interviewers judged that the digital interviews did not differ in content or length compared to the live interviews. Strengths of the study included researcher triangulation. Several researchers were engaged in collecting and analysing the data, which increased the trustworthiness of the findings [38]. In addition, the researchers held continuous reflexive discussions throughout the study [39]. When examining the interview data, it was judged that the data did not suffer from any one participant dominating the discussions. Regarding transferability of the findings, it is possible that the themes are relevant to other individuals who are suffering from Long COVID and are working; however, it has to be recognized that the group is heterogeneous. In qualitative research, it is also meaningful to relate the findings to previous research and thereby add to the accumulation of knowledge [40]. In this paper, the study’s main themes have been discussed in relation to previous research.

## 5 Conclusions

The results from the study, aiming to explore workplace factors that promote and hinder work ability and return to work among individuals with long-term effects of COVID-19, suggest that it is useful to facilitate communication, support and work adjustments. It is also important to accept limitations and fluctuations in work ability and encourage recovery during and after work.

## Ethical approval

This study was approved by the Ethical Review Board, Sweden (2020-05681).

## Informed consent

Informed consent was obtained from all participants with an information letter and the signing of a consent form.

## Conflict of interest

None to report.

## References

[1] Nittas V , Gao M , West EA , Ballouz T , Menges D , Wulf Hanson S , Puhan MA . Long COVID through a public health lens: an umbrella review. Public Health Rev. 2022;43:1604501. doi: 10.3389/phrs.2022.1604501. PMID: 35359614; PMCID: PMC896348 PMC8963488 35359614

[2] NIHR. Living with COVID19: second review (2021) https://evidence.nihr.ac.uk/themedreview/living-with-covid19-secondreview/. Accessed 28 March 2021.

[3] World Health Organization (2021) A clinical case definition of post COVID-19 condition by a Delphi Consensus (2021) https://www.who.int/publications/i/item/WHO-2019-nCoV-Post_COVID-19_condition-Clinical_case_definition-2021.1. Accessed 10 October 2021).

[4] Rajan S , Khunti K , Alwan N , Steves C , Greenhalgh T , MacDermott N , et al. In the wake of the pandemic: Preparing for long COVID. https://apps.who.int/iris/bitstream/handle/10665/339629icy-brief-39-1997-8073-eng.pdf. Accessed 28 March 2021. 33877759

[5] Havervall S , Rosell A , Phillipson M , Mangsbo SM , Nilsson P , Hober S , Thålin C . Symptoms and functional impairment assessed 8 months after mild COVID-19 among health care workers. JAMA. 2021;325(19):2015–16. 33825846 10.1001/jama.2021.5612 PMC8027932

[6] Westerlind E , Palstam A , Sunnerhagen KS , Persson HC . Patterns and predictors of sick leave after Covid-19 and long Covid in a national Swedish cohort. BMC Public Health. 2021;21(1):1–9. 34059034 10.1186/s12889-021-11013-2 PMC8164957

[7] Lemhöfer C , Sturm C , Loudovici-Krug D , Best N , Gutenbrunner C . The impact of post-COVID-syndrome on functioning – results from a community survey in patients after mild and moderate SARS-CoV-2-infections in Germany. J Occup Med Toxicol. 2021;16(1):1–9. 34620202 10.1186/s12995-021-00337-9 PMC8495185

[8] EU-OSHA. Impact of long Covid on workers and workplaces and the role of OSH. https://osha.europa.eu/en/themes/covid-19-resources-workplace. (2022) Accessed 27 May 2022.

[9] Ilmarinen J . Work ability-a comprehensive concept for occupational health research and prevention. Scand J Work Environ Health. 2009;35(1):1–5. 19277432 10.5271/sjweh.1304

[10] Sluiter J , Frings-Dresen M . Quality of life and illness perception in working and sick-listed chronic RSI patients. Int Arch Occup Environ Health. 2008;81(4):495–501. 17638005 10.1007/s00420-007-0222-z PMC2175018

[11] Slebus FG , Kuijer PPFM , Willems J , (HBM, et al.) Prognostic factors for work ability in sicklisted employees with chronic diseases. Occupational and Environmental Medicine. 2007;64:814–9. 17522133 10.1136/oem.2006.031807 PMC2095362

[12] Ladds E , Rushforth A , Wieringa S , Taylor S , Rayner C , Husain L , Greenhalgh T . Persistent symptoms after Covid- qualitative study of 114 “long Covid” patients and draft quality principles for services. BMC Health Serv Res. 2020;20(1):1–13. 10.1186/s12913-020-06001-y PMC7750006 33342437

[13] Kingstone T , Taylor AK , O’Donnell CA , Atherton H , Blane DN , Chew-Graham CA . Finding the ‘right’ GP: a qualitative study of the experiences of people with long-COVID. BJGP Open. 2020;4(5). 10.3399/bjgpopen20X101143 PMC7880173 33051223

[14] Taylor AK , Kingstone T , Briggs TA , O’Donnell CA , Atherton H , Blane DN , Chew-Graham CA . ‘Reluctant pioneer’: a qualitative study of doctors’ experiences as patients with long COVID. Health Expect. 2021;24(3):833–42. 33749957 10.1111/hex.13223 PMC8235894

[15] Barbour RS . Focus groups. In: Bourgeault J Dingwall R de Vries R (eds), The Sage handbook of qualitative methods in health research. Sage, London, (2010), pp. 327–352.

[16] Tracy SJ . Qualitative research methods: collecting evidence, crafting analysis, communicating impact. Wiley-Blackwell, Oxford, (2013).

[17] Ahlstrom L , Grimby-Ekman A , Hagberg M , Dellve L . The work ability index and single-item question: associations with sick leave, symptoms, and health–a prospective study of women on long-term sick leave. Scand J Work Environ Health. 2010;36(5):404–12. 20372766 10.5271/sjweh.2917

[18] Braun V , Clarke V . Using thematic analysis in psychology. Qual Res Psychol. 2006;3(2):77–101.

[19] Giacomini M . Theory matters in qualitative health research. In: Bourgeault I, Dingwall R, de Vries R (eds) The Sage handbook of qualitative methods in health research. Sage, London, (2010), pp. 125–56.

[20] EU-OSHA. (2022) Covid-19 infection and long Covid – guide for managers. https://osha.europa.eu/en/themes/covid-19-resources-workplace. Accessed 27 May 2022.

[21] Aronsson G , Astvik W , Gustafsson K . Work conditions, recovery and health: a study among workers within pre-school, home care and social work. Br J Soc Work. 2014;44(6):1654–72.

[22] Giao HNK , Vuong BN , Tushar H . The impact of social support on job-related behaviors through the mediating role of job stress and the moderating role of locus of control: empirical evidence from the Vietnamese banking industry. Cogent Bus Manag. 2020;7(1):1841359.

[23] Theorell T , Karasek RA . Current issues relating to psychosocial job strain and cardiovascular disease research. J Occup Health Psychology. 1996;1:9–26. 10.1037//1076-8998.1.1.9 9547038

[24] Jonson J , Hall E . Job strain, work place social support, and cardiovascular disease: a cross-sectional study of a random sample of the Swedish working population. Am J Public Health. 1988;78:1336–42. 3421392 10.2105/ajph.78.10.1336 PMC1349434

[25] Mensah A . Job stress and mental well-being among working men and women in Europe: the mediating role of social support. Int J Environ Res Public Health. 2021;18(5):2494. 33802439 10.3390/ijerph18052494 PMC7967617

[26] Halpin SJ , McIvor C , Whyatt G , Adams A , Harvey O , McLean L , et al. Postdischarge symptoms and rehabilitation needs in survivors of COVID-19 infection: a cross-sectional evaluation. J Med Virol. 2021;93:1013–22. doi: 10.1002/jmv.26368. 32729939

[27] Chopra V , Flanders SA , O’Malley M , Malani AN , Prescott HC . Sixty-day outcomes among patients hospitalized with COVID-19. Ann Intern Med. 2021;174:576–478. doi: 10.7326/M20-5661. PMC7707210 33175566

[28] Godeau D , Petit A , Richard I , Roquelaure Y , Descatha A . Return-to-work, disabilities and occupational health in the age of COVID-19. Scand J Work environ Health. 2021;47(5):408. 34003294 10.5271/sjweh.3960 PMC8259700

[29] The work environment 2019. Arbetsmiljöstatistik rapport 2020:2.

[30] Hobfoll SE . Conservation of resource caravans and engaged settings. J Occup Org Psychol. 2011;84:116–22.

[31] Bennett AA , Bakker AB , Field JG . Recovery from work-related effort: A meta-analysis. Journal of Organizational Behavior. 2018;39(3):262–75.

[32] Meijman TF , Mulder G . Psychological aspects of workload. In: Drenth PJD, Wolff CJ (eds). Handbook of work and organizational psychology, vol. 2: work psychology. Psychology Press, Hove, UK, (1998), pp. 5–33.

[33] Graham B , Cottey L , Smith JE , Mills M , Latour JM . Measuring ‘need for recovery’ as an indicator of staff well-being in the emergency department: a survey study. Emerg Med J. 2020;37(9):555–61. 32647025 10.1136/emermed-2019-208797

[34] Nieuwenhuijsen K , Sluiter JK , Dewa CS . Need for recovery as an early sign of depression risk in a working population. J Occup Environ Med. 2016;58:e350–354. 27820770 10.1097/JOM.0000000000000866

[35] van Amelsvoort , LGPM , Kant IJ , Bültmann U , et al. Need for recovery after work and the subsequent risk of cardiovascular disease in a working population. Occup Environ Med. 2003;60(Suppl 1):83i–87i. 10.1136/oem.60.suppl_1.i83 PMC1765716 12782752

[36] de Croon EM , Sluiter JK , Frings-Dresen MHW . Need for recovery after work predicts sickness absence: a 2-year prospective cohort study in truck drivers. J Psychosom Res. 2003;55:331–9. 14507544 10.1016/s0022-3999(02)00630-x

[37] Sluiter JK , de Croon EM , Meijman TF , et al. Need for recovery from work related fatigue and its role in the development and prediction of subjective health complaints. Occup Environ Med. 2003;60(Suppl 1):62i–70i. 10.1136/oem.60.suppl_1.i62 PMC1765724 12782749

[38] Morgan DL , Ravitch SM . Trustworthiness. In: Frey, BB (ed), The Sage encyclopaedia of educational research: measurement and evaluation. 2018.

[39] Malterud K . Qualitative research: standards, challenges, and guidelines. Lancet. 2001;358:483–8. 11513933 10.1016/S0140-6736(01)05627-6

[40] Willig C . Introducing qualitative research in psychology. McGraw-Hill Education, London, 2013.

